# When the Search for Stemness Genes Meets the Skin Substitute Bioengineering Field: KLF4 Transcription Factor under the Light

**DOI:** 10.3390/cells9102188

**Published:** 2020-09-28

**Authors:** Nicolas O. Fortunel, Michèle T. Martin

**Affiliations:** 1 Laboratoire de Génomique et Radiobiologie de la Kératinopoïèse, Institut de Biologie François Jacob, CEA/DRF, Institut de Radiobiologie Cellulaire et Moléculaire, 91000 Evry, France; 2 INSERM U967, 92260 Fontenay-aux-Roses, France; 3 Université Paris-Saclay, 91190 Saint-Aubin, France; 4 Université Paris-Diderot, 75013 Paris, France

**Keywords:** KLF4, adult epidermal keratinocytes, ESC-derived keratinocytes, stemness, skin grafts, TGF-β1, WNT

## Abstract

The transcription factor “Kruppel-like factor 4” (KLF4) is a central player in the field of pluripotent stem cell biology. In particular, it was put under the spotlight as one of the four factors of the cocktail originally described for reprogramming into induced pluripotent stem cells (iPSCs). In contrast, its possible functions in native tissue stem cells remain largely unexplored. We recently published that KLF4 is a regulator of “stemness” in human keratinocytes. We show that reducing the level of expression of this transcription factor by RNA interference or pharmacological repression promotes the ex vivo amplification and regenerative capacity of two types of cells of interest for cutaneous cell therapy: native keratinocyte stem and progenitor cells from adult epidermis, which have been used for more than three decades in skin graft bioengineering, and keratinocytes generated by the lineage-oriented differentiation of embryonic stem cells (ESCs), which have potential for the development of skin bio-bandages. At the mechanistic level, *KLF4* repression alters the expression of a large set of genes involved in TGF-β1 and WNT signaling pathways. Major regulators of TGF-β bioavailability and different TGF-β receptors were targeted, notably modulating the ALK1/Smad1/5/9 axis. At a functional level, *KLF4* repression produced an antagonist effect on TGF-β1-induced keratinocyte differentiation.

## 1. Biomedical Context

**Figure 1:**
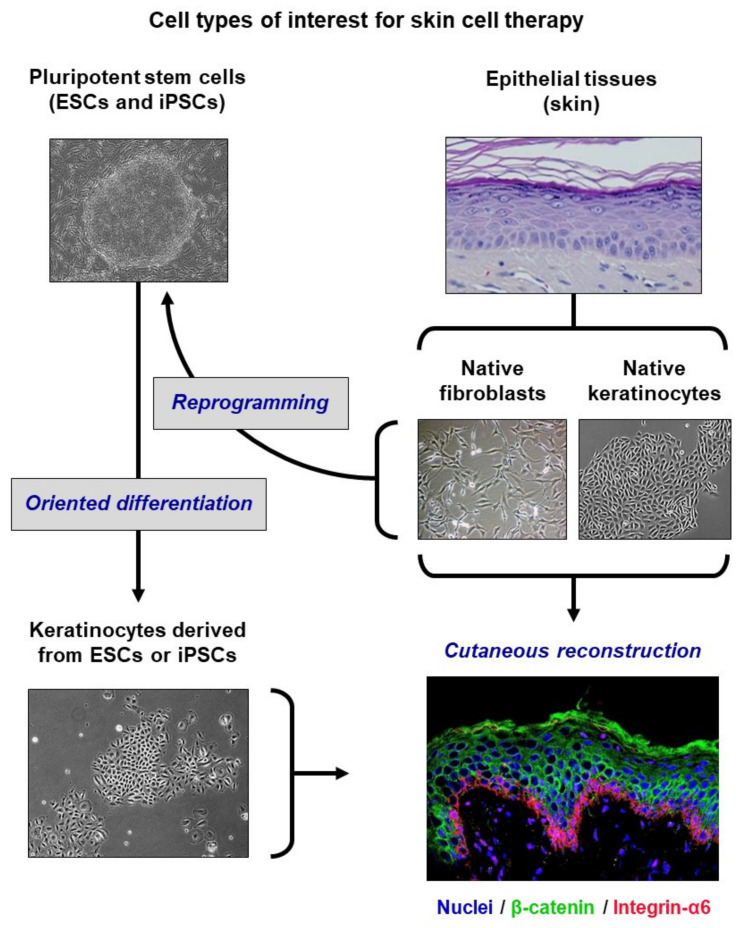
Different sources of keratinocytes of interest for cutaneous reconstruction (adapted from [8]). Native keratinocytes extracted from adult skin biopsies currently constitute the major source of keratinocytes for medical uses. They have been used for more than three decades for the bioengineering of grafts intended for the treatment of severe burns, and more recently for gene therapy. Keratinocytes produced by oriented differentiation of pluripotent stem cells are currently investigated as a complementary source for the development of skin bio-bandages. Embryonic stem cells (ESCs) can be differentiated into keratinocytes capable of generating an epidermis. Pluripotency-induced stem cells (iPSCs), which are obtained by reprogramming adult cells (for example, fibroblasts or skin keratinocytes), can also be differentiated into keratinocytes.

This perspective provides a highlight on a recent publication of our group in Nature Biomedical Engineering [1]. Human epidermis is naturally endowed with remarkable capacities for renewal and regeneration, due to the presence of resident epithelial stem cells within its keratinocyte basal layer. These capacities have enabled the development of different research and clinical models of skin organoids, including skin substitutes that have proved efficient for more than three decades in their use as a treatment for severely burned patients by autologous grafts [2,3]. More recently, an approach combining cell and gene therapies has been successfully achieved to genetically correct the entire regenerated epidermis of an epidermolysis bullosa patient, in the context of a compassionate clinical trial [4]. The bioengineering of large surfaces of skin substitutes, up to one meter squared obtained from skin biopsies of a few centimeters squared, requires a massive ex vivo expansion of patient keratinocytes, during which the preservation of functional stem and progenitor cells is critical to ensure a successful graft take and long-term outcome. Better deciphering the molecular networks that ensure the control of stemness and self-renewal in keratinocytes is thus required for the conception of next-generation skin substitute bioengineering strategies. In addition to native keratinocytes extracted from skin biopsies, keratinocytes produced by the lineage-oriented differentiation of embryonic stem cells (ESCs) may constitute a complementary cell source, as they have the potential to generate three-dimensional (3D) epidermis organoids [5]. Induced pluripotent stem cells (iPSCs) can be obtained by the reprogramming of skin fibroblasts or keratinocytes, from the perspective of re-differentiation towards the keratinocyte lineage (Figure 1). A key input is that autologous iPSCs can thus provide a source of keratinocytes for modeling genodermatoses, such as epidermolysis bullosa, to improve their cellular and molecular characterization, and to test new therapeutic strategies [6,7]. However, the reliability of these alternative keratinocyte sources will depend on the robustness of production methods, and the possibility of obtaining the high proliferative capacity that characterizes native immature keratinocyte stem and progenitor cells, which is not demonstrated today.

## 2. The KLF4 Candidate

The transcription factor “Kruppel-like factor 4” (KLF4) is far from being unknown in the field of stem cell biology. Notably, the KLF family is involved in the regulation of self-renewal and immaturity in ESCs [9], and KLF4 is one of the four factors of the reprogramming cocktail originally described for the generation of iPSCs [10]. In the epidermis, KLF4 is known to exert a regulatory role in keratinocyte terminal differentiation [11], which is essential for the establishment of the barrier function of this epithelium [12]. In contrast, KLF4 functions in native tissue stem cells have been unexplored until now. We performed transcriptomic profiling screens on subpopulations of human keratinocyte precursors, enriched in stem cells or in progenitor cells [13,14] (complete micro-array datasets available in the GEO database, accession no GSE68583), and determined that *KLF4* gene was differentially expressed according to cell immaturity or differentiation. We thus explored the working hypothesis that this transcription factor might regulate epidermal keratinocyte stem cell functions and notably their proliferation and skin regenerative capacity.

## 3. KLF4 Functions in Native Adult Keratinocytes

The regulatory functions of the transcription factor KLF4 have been firstly investigated in “holoclone” keratinocytes [1], which are representative of an immature population of cultured precursors containing functional stem and progenitor cells [15]. These cells correspond to the clonal progeny of single keratinocyte stem cells that were functionally characterized by their extensive growth potential exceeding 100 population doublings through successive subcultures, and the capacity for epidermis reconstruction in vitro and regeneration in vivo. A functional genomics approach was designed to study the effects of *KLF4* repression in holoclone keratinocytes. These cells were transduced with lentiviral vectors designed for “short hairpin RNA” (shRNA)-mediated *KLF4* stably repressed cells, or with a control vector to generate a comparable *KLF4* wild-type cellular context. Using clonal assays and long-term cultures, we demonstrated that maintaining *KLF4* expression at a low level preserves an undifferentiated status in keratinocyte precursors, and promotes their self-renewal. This repression led to the maintenance of high levels of characteristic markers of an immature precursor state, notably integrin α6 and ΔNp63α, and to an improvement of cellular expansion in bidimensional culture. Moreover, the regenerative capacity of *KLF4* stably repressed keratinocytes was higher than that of *KLF4* wild-type keratinocytes. This gain-of-function was demonstrated in vitro by the generation of epidermis organoids, and in vivo by the augmented capacity of *KLF4* stably repressed keratinocytes to ensure iterative xenografting in immuno-deficient athymic nude *Foxn1^nu^* mice [16]. In a first cycle of xenografting, *KLF4* repressed and *KLF4* wild-type keratinocytes were equally potent for regeneration, indicating an absence of deleterious effects of *KLF4* repression on epidermis stratification and differentiation. In contrast, when keratinocytes extracted from primary grafts were tested for a secondary cycle of epidermis reconstruction and xenografting, the success rate obtained with *KLF4* repressed cells was three-fold higher than that obtained with *KLF4* wild-type cells, indicating a better long-term maintenance of functional keratinocyte stem cells in response to *KLF4* repression. The correct differentiation of epidermises regenerated by *KLF4* repressed and *KLF4* wild-type keratinocytes was checked by a histological examination of tissue sections stained with hematoxilin-eosin-saffron (HES), and similar expression patterns of typical epidermis markers such as involucrin, keratin 10 and keratin 5 were observed.

## 4. Insights into the Mechanisms

**Figure 2:**
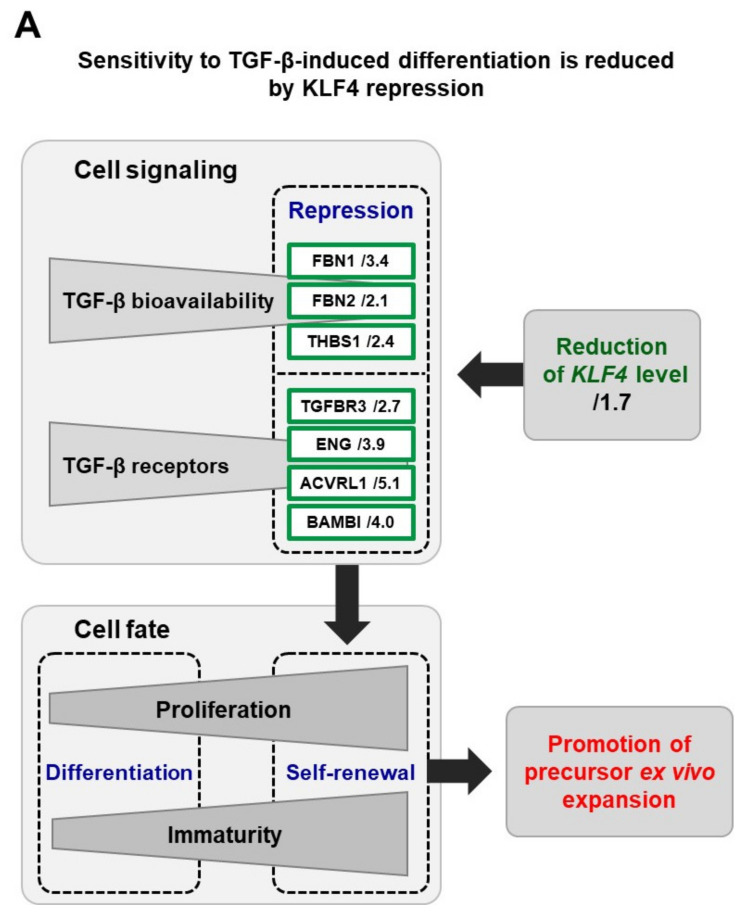

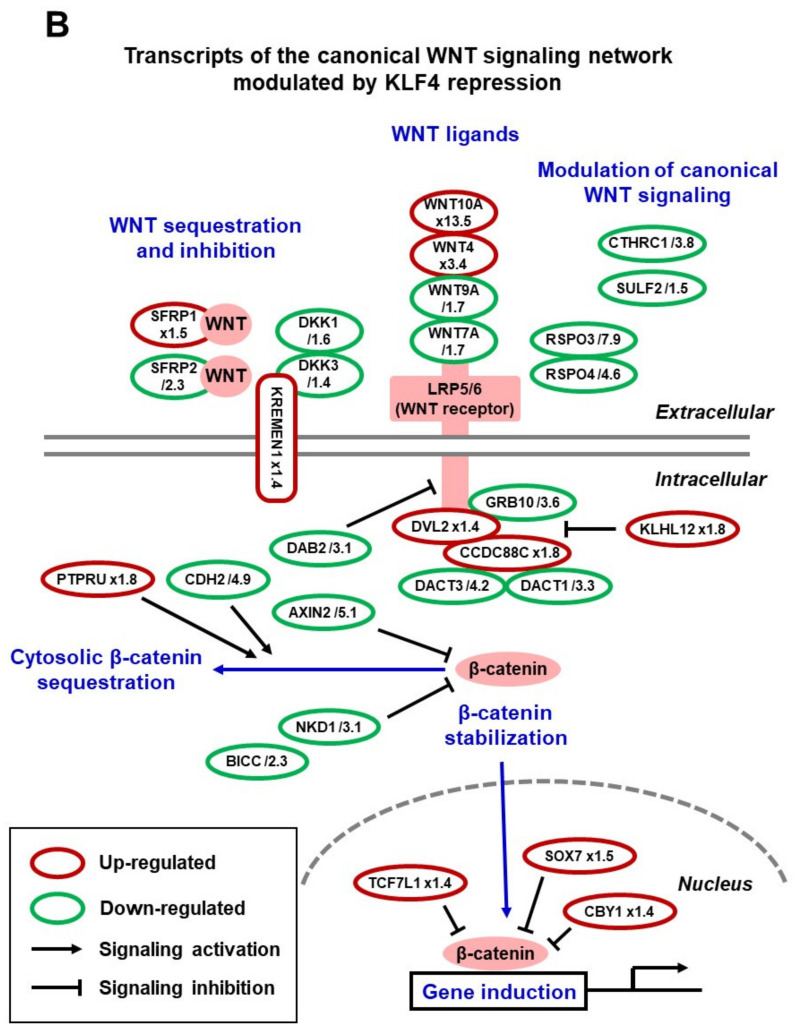
*KLF4* repression impacts the expression of transcripts related to TGF-β and WNT signaling. The comparison of the transcriptional profiles of *KLF4* wild-type and *KLF4* stably repressed keratinocytes, characterized by RNA sequencing (complete datasets are available in the GEO database, accession no GSE111786), showed that the repression of *KLF4* gene expression modulates the expression of numerous genes encoding effectors of the TGF-β (**A**) and WNT (**B**) signaling networks. Notably, a decreased expression was detected for extracellular modulators and membrane receptors of TGF-β signaling. The indicated values correspond to expression fold-changes in *KLF4* stably repressed versus *KLF4* wild-type keratinocytes.

To explain the gain-of-function promoted by *KLF4* repression, a comparative transcriptome profiling of *KLF4* wild-type and *KLF4* stably repressed keratinocyte precursor cells was conducted and analyzed to search for signaling pathways that depend on *KLF4* expression level (complete RNA-seq datasets are available in the GEO database, accession no GSE111786). Analysis of the differentially expressed genes pinpointed the TGF-β1 and WNT signaling pathways (Figure 2), which are major regulatory elements in stem cell biology. A global repression of transcripts related to the TGF-β1 pathway was detected in *KLF4* repressed keratinocytes. Notably, the expression of extracellular modulators of TGF-β bioavailability and of four major membrane receptors of the factor was impaired (Figure 2A) and the ALK1/Smad1/5/9 axis was particularly repressed (Figure 2A). As a known effect of TGF-β1 on keratinocytes is commitment to differentiation [17], the functional relations between this factor and KLF4 were explored. As expected, wild-type keratinocyte precursors entered into differentiation in response to TGF-β1, which was documented by the loss of integrin α6. In contrast, the pro-differentiation effect of TGF-β1 was markedly attenuated in *KLF4* repressed keratinocytes, showing that this repression promotes a better maintenance of stemness. The WNT pathway was also a molecular target of *KLF4* repression, at all regulation levels of the network. As performed for *KLF4*, dedicated functional genomics approaches using RNA interference tools for stable and transient repression will be necessary to elucidate the specific functions of selected WNT candidates pinpointed by our screen (Figure 2B).

## 5. From Basic Research to Pre-Clinical Models

To get closer to a clinically applicable situation, we have then explored the effects of a transitory repression of *KLF4*. Total basal keratinocytes were used [18] as this cellular material is more representative of the clinical samples used for the production of grafts than the research model of holoclones. Firstly, treatment of keratinocytes with “small interfering RNAs” (siRNAs) directed against *KLF4* showed that transient inhibition was efficient at promoting the expression of immaturity-associated markers. In a next step, the feasibility and efficacy of pharmacological KLF4 inhibition was demonstrated using the small molecule kenpaullone, which has been shown to decrease *KLF4* mRNA level [19]. Kenpaullone decreases the *KLF4* level in keratinocyte precursors at both mRNA and protein levels and was efficient to augment their clonogenic capacity and growth in mass cultures. Considering the biosafety issue, exome sequencing did not detect any deleterious effects of kenpaullone treatment on keratinocyte genome integrity. Importantly, reconstructed epidermises generated with kenpaullone-treated cells exhibited normal expression profiles of the classical epidermal markers, laminin 5, integrin α6, filaggrin, and involucrin, showing that kenpaullone treatment did not alter epidermis differentiation. Moreover, using the lucifer-yellow diffusion assay, we could show that epidermises generated by kenpaullone-treated keratinocytes exhibited a lower permeability, compared to those produced with untreated cells, which suggested a more efficient barrier function. The gain-of-function demonstrated by kenpaullone-induced *KLF4* repression may result from a combination of mechanisms. One of them is *KLF4*-mediated attenuation of sensitivity to TGF-β1, as demonstrated in stably transduced keratinocytes. As kenpaullone is also known to target the WNT/β-catenin signaling component GSK-3 [20] at concentrations in the µM range corresponding to those used in our study to repress *KLF4*, modulation of WNT pathway activities can also participate to the observed beneficial effects. Both mechanisms will require further investigations after transient *KLF4* repression.

## 6. Extrapolation to ESC-Derived Keratinocytes

To widen the perspectives that may emerge from the concept of *KLF4* repression in keratinocyte stem cells, the effects of transient *KLF4* downmodulation were also studied on bioengineered keratinocytes obtained by the lineage-oriented differentiation of human ESCs (Ker-ESCs) [1]. Although Ker-ESCs can ensure the reconstruction of correctly differentiated 3D epidermises [5], they do not reproduce all the characteristics of native keratinocytes. Notably, their capacity for proliferation is very limited, probably due to the lack of immature precursor cell status. Kenpaullone also showed efficacy to obtain *KLF4* repression in Ker-ESCs, with a beneficial effect on their proliferative capacity, as described in native keratinocytes. Importantly, kenpaullone-treated Ker-ESCs exhibited a more immature cellular phenotype, which at a functional level resulted in an improvement of growth potential and quality for 3D epidermis reconstruction. In a classical high-density culture condition, untreated and kenpaullone-treated Ker-ESCs equally gave rise to correctly differentiated epidermises, as assessed by a histological examination and visualization of marker expression (involucrin and filaggrin). In contrast, in a stressed condition obtained by low-density seeding, stratification occurred only with kenpaullone-treated Ker-ESCs.

## 7. Conclusions

We identified the transcription factor KLF4 as a regulator of the balance between immaturity and differentiation in the human keratinocyte lineage, which is set notably via interactions with the TGF-β1 signaling pathway. One mechanistic interpretation of our data is that keratinocyte precursor cells exhibiting *KLF4* repression are less sensitive to TGF-β1-mediated commitment to differentiation, through a reduction in TGF-β1 extracellular bioavailability and an impairment of its cell-surface receptor machinery. At the basic research level, further deciphering these molecular links will be needed to elucidate the related mechanisms. More generally, the molecular networks and cascades involved constitute an elegant model for further dissecting the molecular identity of stemness [21,22]. In addition, our study suggests that interactions between KLF4 and WNT signaling also participate in the regulation of epidermal precursor and stem cell proliferation and immaturity maintenance during in vitro amplification (Figure 2B). A major objective is the full dissection of the specific roles of KLF4 in the different epidermis layers. In the upper part of mouse epidermis, KLF4 has been shown to be essential for keratinocyte terminal differentiation and barrier function [12]. In a human model, such an induction of differentiation genes has been shown to be driven notably by interactions between KLF4 and epigenetic factors such as chromatin modifiers and long non-coding RNAs [23,24,25]. We propose that KLF4 might be a gate keeper of stemness in the basal layer of epidermis, thus exerting different roles through specific partners in different epidermis layers. A recent study performed on N/TERT-immortalized human keratinocytes and mouse skin has shown that regulatory loops involving KLF4 together with the YAP1/TAZ-TEAD transcriptional network participate to the control of the equilibrium between proliferation and differentiation [26]. In these models, YAP1/TAZ-TEAD promote keratinocyte proliferation, while KLF4 drives cells towards differentiation, which is in agreement with our finding that lowering KLF4 level improves the proliferative capacity of immature keratinocyte precursor cells.

**Figure 3:**
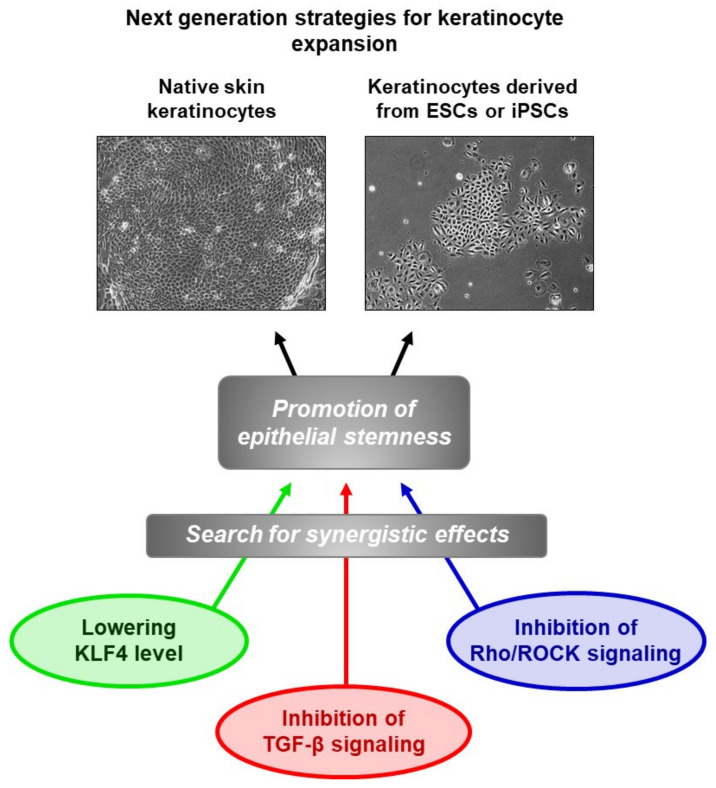
Improvement of keratinocyte ex vivo amplification using molecules promoting epithelial stemness is currently an active research field. The work of our laboratory has identified the transcription factor KLF4 as a relevant target, which could be used in combination with other candidate targets, in order to develop culture media of defined composition for biomedical uses.

At the translational research level, the key finding that emerged from the study is that maintaining KLF4 at a low level by transient pharmacological repression constitutes a promising approach to promote the ex vivo expansion of two types of cells of interest for cutaneous graft bioenginering: native epidermal keratinocytes and ESC-derived keratinocytes. Moreover, the ex vivo expansion of keratinocytes for clinical purposes is generally performed in undefined culture media containing bovine serum in the presence of growth-arrested feeder fibroblasts [27]. This situation is expected to progressively evolve towards undefined and animal-component-free culture conditions to avoid the risk of introducing unknown pathogens or any kind of deleterious biomolecules. Considering this demand of regulatory agencies, the substitution of undefined culture components by active molecules promoting stemness, such as KLF4 inhibitors, will open original perspectives for the development of next-generation models of skin substitutes. In this context, the use of PAK1-ROCK-Myosin II and TGF-β signaling inhibitors has been proposed to promote the preservation of stemness in expansion cultures of different types of epithelial cells [28]. Clues will certainly emerge from the combination of these converging studies (Figure 3).
